# Post-intervention perceptions on the antiretroviral therapy community group model in Trans Nzoia County, Kenya

**DOI:** 10.11604/pamj.2024.47.113.41843

**Published:** 2024-03-08

**Authors:** Violet Naanyu, Hillary Koros, Suzanne Goodrich, Abraham Siika, Cathy Toroitich-Ruto, Moses Bateganya, Kara Wools-Kaloustian

**Affiliations:** 1 Department of Sociology Psychology and Anthropology, School of Arts and Social Sciences, Moi University, Eldoret, Kenya,; 2 AMPATH Qualitative Research Core, Academic Model Providing Access to Healthcare, Eldoret, Kenya,; 3 Division of Infectious Diseases, Indiana University School of Medicine, Indianapolis, IN, USA,; 4 Department of Medicine, School of Medicine, College of Health Sciences, Moi University, Eldoret, Kenya,; 5 Division of Global HIV and TB (DGHT), Centers for Global Health (CGH), US Centers for Disease Control and Prevention (CDC), Nairobi, Kenya,; 6 Family Health International fhi360.Org, North Carolina, USA

**Keywords:** Community-level care, HIV care, focus group discussions, peer-based care, Kenya

## Abstract

**Introduction:**

the increasing number of people receiving antiretroviral therapy (ART) in sub-Saharan Africa has stressed already overburdened health systems. A care model utilizing community-based peer-groups (ART Co-ops) facilitated by community health workers (CHW) was implemented (2016-2018) to address these challenges. In 2018, a post-intervention study assessed perceptions of the intervention.

**Methods:**

forty participants were engaged in focus group discussions consisting of ART Co-op clients, study staff, and health care providers from Kitale HIV clinic. Data were analyzed thematically for content on the intervention, challenges, and recommendations for improvement.

**Results:**

all participants liked the intervention. However, some reported traveling long distances to attend ART Co-op meetings and experiencing stigma with ART Co-ops participation. The ART Co-op inclusion criteria were considered appropriate; however, additional outreach to deliberately include spouses living with HIV, the disabled, the poor, and HIV pregnant women was recommended. Participants liked CHW-directed quarterly group meetings which included ART distribution, adherence review, and illness identification. The inability of the CHW to provide full clinical care, inconvenient meeting venues, poor timekeeping, and non-attendance behaviors were noted as issues. Participants indicated that program continuation, regular CHW training, rotating meetings at group members´ homes, training ART Co-ops leaders to assume CHW tasks, use of pill diaries to check adherence, nutritional support, and economically empowering members through income generation projects would be beneficial.

**Conclusion:**

the intervention was viewed positively by both clinic staff and clients. They identified specific challenges and generated actionable key considerations to improve access and acceptability of the community-based model of care.

## Introduction

To preserve the health of individuals living with HIV and to curb the HIV epidemic, the 2016 World Health Organization (WHO) consolidated guidelines on the use of antiretroviral drugs for treating and preventing HIV infection and recommended antiretroviral treatment (ART) for everyone living with HIV regardless of their CD4 cell count [1]. This recommendation has increased the burden on already strained health care systems in sub-Saharan Africa. Financial constraints and logistical issues impede regular monitoring of clients taking ART [2,3] and problems with provider-patient interaction and communication have been a challenge to optimizing ART delivery [4,5]. HIV care can also present a logistical burden for persons living with HIV and their families. Long distances to service points, unreliable and costly transportation, and long wait times in clinics are often barriers to optimal adherence and retention in care [6-9]. Lay volunteer health care workers have facilitated adherence within health clinics and in the community [10,11]. Peer educator programs have also been used to assist clients in navigating care and providing health education, social support, and counseling [12]. Community-level use of people living with HIV (PLHIV) trained to distribute ART and provide adherence counseling has been studied previously. For instance, in Mozambique, community ART groups have PLHIV as active participants in their own care and the care of others [13,14]. The ART Co-ops intervention described elsewhere [15,16] was implemented in the period 2016-2018 to decrease the strain on the clinic infrastructure, as well as the time and cost burden to clients. The ART Co-ops intervention study was a geographically randomized control trial of standard of care ART services in the clinic versus quarterly CHW-facilitated group care within community-based groups. At the end of the one-year intervention period, we conducted a qualitative assessment of the ART Co-ops intervention to better understand the acceptability of the intervention, as well as to gather recommendations about how the intervention could be improved. This manuscript is only focused on the end line qualitative assessment.

## Methods

**Study design:** this study was a cross-sectional, post-intervention, qualitative evaluation that utilized focus group discussions (FGDs) with clients, health care providers (HCPs), and the research study staff in the ART Co-ops study.

**Study setting:** this study was conducted in January 2018 in Trans Nzoia County, Kenya. It was based at the Academic Model Providing Access to Healthcare (AMPATH) clinic in Kitale, a HIV care and treatment program currently operating in western Kenya [17]. At the time of initiating the ART Co-op intervention (in 2016), six clinical officers (CO), six nurses and several ancillary staff provided care at the clinic. Stable clients were seen every 1-2 months, with a CO visit scheduled for a minimum of every 6 months and a maximum of monthly. By 2016, the clinic had over 19,000 clients enrolled, with >12,000 on ART. This was one of AMPATH´s most rapidly growing clinic populations and served a large geographic area, which made it an ideal site for studying and implementing the use of a community care model.

**Study participants:** forty participants were involved in six FGDs. The first FGD included HCPs (two men, and four women) who were serving patients at the Kitale AMPATH HIV clinic, available during the study period, and were willing to participate in the FGD. The second FGD included personnel from the study, i.e., four CHWs, the study´s clinical officer, and the study´s site coordinator. The remaining four FGDs were held with randomly selected adult clients from the ART Co-ops intervention. As ART Co-ops members began resuming care at the Kitale clinic, every third patient was invited to participate in the FGDs. The client FGDs were divided by sex (2 for women and 2 for men), with each of the women´s groups having seven participants, and the men´s groups having six and eight participants, respectively.

**Variables:** the FGD sessions were moderated through the use of a question guide that had three main domains (Annex 1). The first domain addressed participants´ understanding of the ART Co-ops intervention components. They included: 1) the purpose of developing ART Co-ops; 2) eligibility criteria to participate in a Co-op group; 3) group meeting times; and, 4) group direction by a Community Health Worker (CHW). The second domain asked participants to evaluate each aspect of the intervention and share opinions on the intervention. The third domain asked participants to share their views on how the intervention had motivated HIV care uptake and adherence. It also solicited recommendations to improve the intervention.

**Data collection:** a moderator and scribe facilitated each of the six FGDs in a private space identified at the Kitale AMPATH clinic. There were three experienced (one male, 2 female) research assistants with health or social sciences diplomas. They received an intensive training on the study background, study tools, and consent procedures. They also got refresher training on research ethics, FGD methodology, and data management and storage during fieldwork. Participants were asked to share their perceptions of the ART Co-ops model. The discussions were in English and Kiswahili according to participants´ preferences. Open-ended interview guides were used to facilitate the FGDs (Annex 1). The FGDs were on average one hour long, and they were audio-recorded and later transcribed for analysis. All participants provided written consent.

**Data analysis:** the audio recordings were transcribed verbatim and translated to English where necessary. NVivo version 12 was used to manage and analyze data. Two coders used both inductive and deductive coding to co-develop the coding framework. All transcripts were first coded by one coder and then reviewed by the second coder. Where there were discrepancies, the two coders jointly reviewed relevant codes and data and came to an agreement. Subsequently, they identified relevant thematic categories for analysis in line with the study questions. Findings are presented in four broad areas: 1) opinions on the organization of the ART Co-op intervention; 2) positive valuation of the intervention; 3) thoughts on shortcomings of the intervention; and 4) recommendations regarding the ART Co-ops intervention. Illustrative quotes were also selected to exemplify study findings (Table 1). Reporting on this study was guided by COREQ guidelines and STROBE checklist [18].

**Table 1:** perceptions on the ART co-ops model facilitated by community health workers, Trans Nzoia, Kenya

	Specific code	Illustrative excerpts
**Positive valuation**	Convenience of care at community level	“Where I come from, the community is of the opinion that this ART Co-op project should continue… when the services are nearer, people are able to resume to their works as fast as possible.” (Female participant, FGD 2)
	Reduced transport costs	“I am also happy to be part of the group because we saved time and the transport cost has reduced… here we can even walk, take a short time to be attended to, finish up early and get back home.” (Male participant, FGD 2)
	Efficient and empathetic CHWs	“CHWs are good, they have made us feel like we are no longer sick, they talk to us well and in a soothing way…unlike in the previous clinic where we were being shouted at most of the time. Again, they keep time, you are served with the shortest time possible and you are back home early… Our CHWs …make you feel you are a worthy person. This is unlike in the past, where we were talked to so harshly and in very intimidating ways.” (Male participant, FGD 1)
	Reduced time needed for care	“I personally feel it was so beneficial…unlike in the past when we used to get out of the house at 6am and back at 6pm. We queued for long hours… But now that we are in groups, things have become better.” (Female participant, FGD 1)
	Promoted adherence and retention to care	“For me, it has helped in line with drug adherence because you know… the CHW will ask how you have been using your drugs. He will count to know if you’ve missed any in that duration. If I have missed, I just say the truth and he will correct me accordingly and that is something that has impressed me a lot…” (Female participant, FGD 2)
	Reduced congestion	“For us clinician we have ample time with our clients...It is not congested…So we take time to see the patient.” (HIV Clinic Clinicians)
	Opportunities for socio-economic activities	“The beauty of the groups we formed was that we understood each other well… We have even formed a “merry go round” and now we are interacting more like sisters.” (Female participant, FGD 1)
	Reduced HIV related stigma	“It has also reduced stigma to the patients, because unlike coming here [HIV clinic] …when meeting in the community, they will just say something else but coming here, people will be sure that they will be coming for [HIV] help.” (CHWs and Study staff)
**Shortcomings**	Long distances to some group meetings	“There are those clients who are still using transport/fare coming to the group and which was still costly for them. We felt like we were not yet going closer to the community.” (CHWs and Study staff)
	CHWs offered limited services	“I also think it was not complete package in terms of medical care because when a client goes to the facility, if there is need for her to see a nutritionist, or a social worker, it is easy. But in the community, it is just getting medication and maybe some counseling… they feel like you have not given them all the health care they need…” (CHWs and Study staff)
	Potential for increased stigma	“As much as it reduced stigma in a way, it has also increased it. For instance, when we meet in someone’s home…there were others who were afraid of the other neighbors… ‘What if … they want to know why we are meeting?’ Because there are nosy neighbors who will come into that house and then start asking funny questions.” (CHWs and Study staff)
	Poor time keepers	“There are people who don’t observe time, keeping the CHW waiting for long, yet he is the one to benefit.” (Male participant, FGD 1)
	Non-attendance	“I can say, all members are attending the meetings except one who always lags behind. When the CHW calls him, he says he will come later.” (Male participant, FGD1)
	Limitations in meetings venues	“Then the group had just started, we were stationed at [X] health centre. The health center community felt like ‘we’ the AMPATH group were intruding on their own clinics…At some point we felt like there was a need to change the venue but we got used to their back bites and moved on…” (Female participants, FGD1)

**Ethical considerations:** the study was reviewed and approved by the Moi Teaching and Referral Hospital and Moi University College of Health Sciences institutional ethics committee (IREC # 0001287) the Indiana University Institutional Review Board (IRB #1409002670), and was also approved by relevant County and health facility leadership. The study was also reviewed in accordance with the U.S. Center for Disease Control and Prevention (CDC) human research protection procedures and was determined to be research, but the CDC investigators did not interact with human subjects or have access to identifiable data or specimens for research purposes.

## Results

Generally, study findings show all participants positively evaluated the intervention and shared ideas on areas of improvement (Table 1). Participants reported that the intervention had motivated people to take care of their HIV condition and based on their positive experiences at the ART Co-ops, they encouraged other PLHIV to seek clinical care.

**Organization of the ART Co-op intervention:** participants described the ART Co-op inclusion criteria as clear, and those who were eligible were 'seen as lucky´ to have been engaged. The ART Co-op group meetings were held every three months, and participants were satisfied with this arrangement. It was more convenient for them than going to the HIV clinic, which was far from where they lived. ART Co-op participants felt less time pressure in attending to their HIV care - they could easily complete other daily activities, even on the same day that they needed to go for care. All FGDs reported that the CHWs performed their roles well. CHWs competently kept track of group meetings and followed up with clients. All FGDs noted the CHW ensured faster service than the usual standard of care at the HIV clinic. In five of the six FGDs, participants reported that the CHWs demonstrated confidence, politeness, and patience. They were organized, punctual, confidential, understanding, and concerned about their clients. In three FGDs (HCP, study staff, and men´s FGD 1), participants noted that CHWs were appreciated in their communities.

**Positive valuation of the intervention:** overall, participants expressed satisfaction with the ART Co-ops intervention (Table 1). Advantages associated with the ART Co-ops are summarized into eight sub-categories: convenience of care at the community level, reduced transport costs, efficient and empathetic CHWs, reduced time needed for care, promotion of adherence and retention in care, reduced congestion at the health facility, opportunities for socio-economic activities, and reduced HIV-related stigma. All FGDs noted the convenience of community-based services because it reduced transportation costs and time away from their businesses/chores. All participants positively viewed the use of CHW to identify those in need of clinical care and make referrals when necessary. Five FGDs (HCP FGD, study staff FGD, two women´s FGD, and men´s FGD 1) reported that the CHW services were easily available and physically accessible to the participants. According to two FGDs (study staff FGD and women´s FGD 1), clients were glad to have the CHW check their vital signs and quickly provide drug refills. In all FGDs, participants felt that pill counts by the CHW were a good way to evaluate and encourage adherence. Five FGDs (HCP and all patient FGDs) believed the intervention spared them the long queues endured by clinicians and clients at the health facilities. Four FGDs (HCP, two women´s FGDs, and one men´s FGD) discussed how CHWs enjoyed a sociable relationship with clients and CHWs were described as having a positive attitude. According to participants in four FGDs (Women´s FGD 1, HCP, study staff, and two men´s FGDs), engagement of CHW in this intervention increased patients´ comfort during care because compared to regular clinician-patient encounters. ART Co-op participation provided opportunities for joint social and income generation prospects. Group interactions fostered camaraderie and a deeper understanding of each other´s conditions. Lastly, four FGDs (HCP, study staff, and two women´s FGDs), reported that the intervention reduced HIV-related stigma experienced by clients attending care at the HIV clinic. Group meetings within the community could be inconspicuous and assumed to be part of any social activity, while clinic visits were associated with receiving HIV-related services - a potential opportunity for social stigma.

**Shortcomings of the ART Co-ops intervention:** disadvantages participants associated with the ART Co-ops are summarized into six sub-categories (Table 1): long travel distances to some group meetings, limited CHW-offered services, potential for increased stigma, poor time-keeping, inconsistent member attendance, and limitations in meeting venues. According to three FGDs (study staff, women´s FGD 1, and men´s FGD 2), some group members still covered long distances to attend meetings and attendance worsened during rainy seasons when means of transport were limited. Two FGDs (HCP and study staff) noted that CHWs were unable to offer comprehensive services as ART Co-op´s services were limited to vital sign assessment, a general check for any bodily difficulties, some counseling, and drug refills. Study staff participants reported that some people feared the possibility of increased stigma through involvement in community groups. They argued that, although the group model reduced some stigma, there was potential for inadvertent disclosure and social stigma as meetings occurred in the community. Poor timekeeping was noted by two FGDs (men´s FGD 1 and women´s FGD 2) and HCP emphasized that some members failed to justify their non-attendance. Participants from four FGDs (HCP, two women´s FGDs, and men´s FGD 2) reported difficulties getting convenient meeting venues.

**Considerations for improvement of the intervention:** participants shared changes that could improve the ART Co-ops model. Regarding inclusion criteria, they felt the ART Co-ops should purposively recruit spouses with HIV, people living with disabilities (PLWD), the poor, very busy working individuals, and pregnant women. There were suggestions about changing the frequency of the Co-ops meetings, with three FGDs (study staff and two women´s FGDs) proposing decreasing the frequency of meetings from every three months to every four or six months. In all FGDs, participants said CHWs were already known for providing health services in the communities, and two FGDs (HCP and study staff) thought CHWs could receive additional training to more ably support patient care. A range of other individuals were considered for leadership of the ART Co-ops including any trustworthy group members (two women´s and one men´s FGD); nutritionists, nurses, pharmacists, and other clinicians (HCP FGD); and psychosocial workers living with HIV (study staff). Participants also thought peer group leaders could replace CHVs in the distribution of ART. In four FGDs (HCP, study staff, and one women´s and one men´s FGD) participants recommended the use of pill diaries to assess adherence. The HCP FGD also suggested assessment of and attention to the client´s nutritional status. Overall, there was interest in having the ART Co-ops program continue and expand. To facilitate this, participants suggested increasing community sensitization to the intervention. In addition, they suggested that the peer groups be formally recognized and registered, and empowered with training on income generation projects.

## Discussion

This post-intervention study aimed to understand the perceptions of HCP, study staff and participants of a community-delivered ART model provided in Trans Nzoia County. Overall, both HCP and participants were very positive about the model and provided ideas about how the model could be improved to reach and better serve more PLWH. The positive view of the ART Co-ops model as a facilitator to HIV care uptake and adherence has been reported in past studies [19-23]. The ART Co-ops model reduced transport costs and time spent on health care. PLWH enjoyed the convenience of care at the community level and away from the congested health facilities. The use of the ART Co-ops is likely to reduce congestion at the health facility. Staff shortages at health facilities coupled with long client queues can lead to burnout and poor quality of care [24,25]. Decentralizing the care through a community model such as the ART Co-ops provides an accepted and realistic option for this problem. Earlier studies have shown the value of CHW in addressing human resource shortages in HIV care in Africa [26,27]. The community model addresses clinic congestion issues and matches CHW-facilitated meetings to PLWH needs and availability, with a focus on quality service delivery and peer support. Community-based care in support of decongestion of service delivery for PLWH [12] is desirable, especially in the context of COVID-19, where physical distancing or social isolation are recommended to reduce the spread of COVID-19 [28,29].

Consistent with other studies, health care delivery models that address the need for geographical accessibility are likely to be embraced, especially in rural economies [30]. The ART Co-ops reduce transport costs and mitigate budget concerns. A cross-sectional analysis of ART coverage in the 47 counties of Kenya established that poverty was the most critical barrier to ART [31]. In the face of financial constraints, clients struggle to decide whether to spend the little money available on school fees and food, or on transport to health facilities, which is at times exacerbated by long distances to the clinics [32]. Our study adds to the literature on the value of community-level HCWs and peers in enhancing adherence to HIV care [10,11,13,14,22,26,27]. Looking at past studies, the use of peer group leaders as part of a care delivery team has supported ART programs whereby clients learn and grow together, and develop personal connections that are important to patients and their health care [33,34]. Our findings found that the use of empathetic CHWs who serve as a peer group for PLWH was seen as an advantage of the community care model. Similar to past studies, engagement of CHWs increases clients´ trust, comfort, and personal connectedness [35]. The supportive CHW-client interaction is valued and bridges a gap where provider-client interactions have been known to be poor [22]. It is prudent to consider these approaches in Kenya and similar low-middle-income countries (LMICs) to promote adherence and in care.

As supported by our study, earlier studies demonstrate HIV stigma hampers HIV care uptake activities [23,32,36,37]. The ART Co-ops model had the potential to reduce (e.g. through peer support and localized care) and also increase HIV-related stigma (e.g. through social stigma in their home environment). Care at the community level seems to reduce opportunities for HIV-related stigma documented in past studies in HIV clinic settings [23,36,38]. In this study, the ART Co-ops provided opportunities for peer bonding and even prospects for shared socio-economic activities. This highlights the importance of providers and beneficiaries of community-based care models understanding the local context and intervention components that may exacerbate self and social stigma. Reported shortcomings of the model included long distances to some group meetings, poor time-keeping, inconsistent attendance, challenges locating convenient meeting venues, limited CHW-delivered services, and the potential for increased social stigma. Challenges are inevitable when setting up community-based ART programs and should be anticipated in the design of interventions. When developing such models, a human-centered design can be used to ensure a contextual understanding of the local situation, and consequent development of an ART model through iterative prototyping, end-user engagement, and usability testing [39]. In this study, participant-suggested changes to the ART Co-op model include increased community sensitization to the model, engagement of more CHWs, and formal registration and training on income generation projects. The inclusion of spouses, PLWD, the poor, very busy working individuals, and women in PMTCT programs was suggested. Nutrition support, use of pill diaries to check adherence, fewer group meetings, consideration of additional cadres to serve as group leaders and use of peers as ART distributors were all recommended. As supported by other findings in the region [23,40], study participants identified the need for nutritional assessment and support for PLWH. The perceived need to consume a “special diet” while on ART fuels a sense of food insecurity when patients fear their needed diet may not be sustainable [41], especially if the patients are unemployed [42]. Appreciation of local circumstances and the basic needs of relevant community members is an important element of planning intervention programs.

**Strengths and limitations:** this paper provides findings from a cross-sectional, post-intervention, qualitative assessment of the ART Co-ops model. A fuller exploration of the experiences of all who were involved in the intervention model - from initiation to its conclusion, would provide a more comprehensive lens on community-level models of care. The intervention was implemented in Trans Nzoia, Kenya, and this study only includes reports of people who had been directly involved in the intervention: PLWH, HCW, and the research study staff. This study only engaged PLWH who remained in the community model - there was no follow-up of any patients who may have joined the ART Co-ops and then dropped out. As such, the findings reflect the views of only those who were retained. Our findings may not be representative of other community-level models of care. Further studies are needed to more rigorously examine ART Co-op models and their effectiveness for ART delivery. Despite these limitations, this study expands our understanding of community-based ART programming and associated challenges, especially in the Kenyan context. Our study provides information on the acceptability of community-level models of ART and offers insight into possible ways to improve access and acceptability of such models of care.

## Conclusion

Innovative care delivery models are needed to ensure the provision of ART becomes a reality in resource-challenged settings. The ART Co-ops model sought to reduce barriers to ART by making care more accessible. This post-intervention assessment shows support for this care option while identifying its weaknesses and actionable plans for improvement. Given the clear appreciation for the model, additional research can explore the use of ART Co-ops in other settings. More assessments can compare community models to three-month and six-month medication dispensing for HIV patients, including assessing uptake of service, treatment outcomes, costs, and stakeholder perspectives on the care. Furthermore, more studies can assess how well other community-level and peer-based care models serve clients living with diverse health conditions.

### What is known about this topic

- *Globally, there is an increase in the number of patients receiving ART, however, HIV care presents logistical burdens for people living with HIV;*
- *Lay health care workers and peer educators have capably facilitated adherence within health clinics, and in select community settings*.

### What this study adds

- *This study shows that community-level and peer-based care models of ART can be feasibly implemented in resource-challenged settings in Kenya;*
- *This study shows perspectives of diverse end users are valuable in assessing innovative ART models and their possible improvements for further reach and acceptability*.

## Annex

**Annex 1**: focus group discussions: post-Intervention perceptions on the ART community group model (PDF-538 KB)
